# Parental Distress in the Time of COVID-19: A Cross-Sectional Study on Pediatric Patients with Neuropsychiatric Conditions during Lockdown

**DOI:** 10.3390/ijerph18157902

**Published:** 2021-07-26

**Authors:** Gianluca Sesso, Eleonora Bonaventura, Bianca Buchignani, Stefania Della Vecchia, Caterina Fedi, Marisa Gazzillo, Jessica Micomonaco, Andrea Salvati, Eugenia Conti, Giovanni Cioni, Filippo Muratori, Gabriele Masi, Annarita Milone, Roberta Battini

**Affiliations:** 1 Department of Clinical and Experimental Medicine, University of Pisa, 56126 Pisa, Italy; gianluca.sesso@fsm.unipi.it (G.S.); eleonora.bonaventura@fsm.unipi.it (E.B.); bianca.buchignani@fsm.unipi.it (B.B.); stefania.dellavecchia@fsm.unipi.it (S.D.V.); caterina.fedi@fsm.unipi.it (C.F.); marisa.gazzillo@fsm.unipi.it (M.G.); jessica.micomonaco@fsm.unipi.it (J.M.); andrea.salvati@fsm.unipi.it (A.S.); giovanni.cioni@fsm.unipi.it (G.C.); filippo.muratori@fsm.unipi.it (F.M.); 2 Department of Developmental Neuroscience, IRCCS Stella Maris Foundation, 56128 Pisa, Italy; eugenia.conti@fsm.unipi.it (E.C.); gabriele.masi@fsm.unipi.it (G.M.); annarita.milone@fsm.unipi.it (A.M.)

**Keywords:** COVID-19, children, adolescents, parental stress, neuropsychiatric disorders

## Abstract

The lockdown due to the COVID-19 pandemic has had adverse psychological effects on children and parents. While parenting is essential for positive development, increased parental distress has interfered with children’s wellbeing. In our study, we aimed to identify the predictors of parental distress in families of children with neuropsychiatric disorders during lockdown. Seventy-seven parents of children with neuropsychiatric disorders were asked to fill three online questionnaires (a socio-demographic questionnaire, the Child Behavior Checklist (CBCL) and Parental-Stress-Index (PSI-4-SF) to explore the relationship between parental distress, emotional/behavioral problems in children and quarantine-related factors through univariate analyses and multiple mediation models. Significant positive associations between CBCL-internalizing-problems and all PSI-4-SF subscales, and between CBCL-externalizing-problems and “Difficult Child” subscales were found. “Parent–Child Dysfunctional Interaction” subscale and teachers–child relationship quality resulted negatively associated, as well as the “Difficult Child” subscale and peers–child relationship quality. The effect of teachers–child relationship quality on “Parent–Child Dysfunctional Interaction” was mediated by children internalizing problems, while the effect of peers–child relationship quality on “Difficult Child” by the child internalizing/externalizing problems. Internalizing problems in children with neuropsychiatric disorders were among the strongest predictors of parental stress during lockdown, mediating the indirect effects of quarantine-related factors, thus suggesting the importance of their detection during and after emergency situations to provide assistance and reduce parenting pressure.

## 1. Introduction

Parenting stress is a psychological reaction that parents may experience when parental expectations are not met or are unsustainable by the parents themselves [1,2]. It is conceptually distinct from other forms of distress that parents might experience, such as, for instance, financial hardships, work stress or negative life events, though stress associated with parental duties and other life stressors are also frequently associated [1]. A strong relationship has been repeatedly confirmed between parenting stress and family and individual emotional outcomes, including parental mental health and wellbeing [3], offspring emotional and behavioral issues [4], child academic achievement [5], parenting behaviors [6], parent–child relationship quality [1] and family functioning [2].

During the recent lockdown imposed by the Italian government between March and June 2020 to contain the spread of the infectious disease due to SARS-CoV-2 (or coronavirus disease 2019 (COVID-19)), the role of parents and caregivers at home has become essential, being the only direct educational model for most children. Indeed, the daily lives of children and their families rapidly changed, and schools, educational support services and sports or recreational activities were all interrupted and replaced by Internet-based video-lessons and distance learning [7]. Meanwhile, most parents carried on their jobs and professional activities during the lockdown, however, in newly experienced smart-working formats, which were far from being compatible with full-time parenting duties. Moreover, many parents also worried about the financial hardships caused by lockdown measures and the health issues of their elderly or other family members [8,9]. Indeed, a considerable number of recent studies revealed a substantially increased risk of distress and negative emotional outcomes in parents during lockdown [10,11,12,13,14,15]. Interestingly, not only biological parents but also foster parents reported significant increases in parenting stress, especially those who were not married or reported poorer mental health or disadvantageous financial circumstances [13]; moreover, among parents, those who worked in health care facilities lived with a chronic physical illness or had acquaintances diagnosed with COVID-19 displayed significantly higher levels of parental distress [14,16]. Finally, younger age and the gender (woman) of parents were independently associated with greater parenting stress [14].

While optimal parenting is considered essential for positive development, increased parental distress could potentially interfere with children wellbeing.

Interestingly, Spinelli and colleagues (2020) [17] recently investigated parental distress risk factors during the COVID-19 outbreak and its impact on offspring mental health in a population of 2 to 14 years old children and their families which were recruited with an online survey. The authors revealed that the impact of quarantine on children’s emotional regulation strategies and their negative emotionality was mediated by both parental stress and the degree of parents’ involvement with their offspring [17]. In this study, parenting stress was investigated through the Parent/Child Dysfunctional Interaction (PCDI), a subscale of the Italian short version of the Parenting Stress Index—short form (PSI-SF) questionnaire, a scale that has been widely used to measure parenting stress in studies conducted on both clinical and high-risk populations [18,19,20]. In addition, general young population, children with special needs or disabilities and their families reported severe psychological lockdown-related consequences [8]. Purposefully, our recent study [9] was specifically aimed at exploring emotional and behavioral consequences of a COVID-19 outbreak in a population of children and adolescents affected by neurological and psychiatric conditions. Particularly, our findings revealed an increase in anxiety and somatic problems in preschoolers aged 1.5–5 years, while obsessive compulsive symptoms, post-traumatic and thought problems were higher in children and adolescents aged 6 to 18 years [9]. Moreover, younger age was found to be a protective factor in increasing the psychiatric symptom severity, while the financial hardship experienced by families during lockdown worsened it [9].

However, only few studies to date have investigated the impact of COVID-19 quarantine on perceived distress in parents of children with neuropsychiatric conditions. Pasca and colleagues (2020) [21] assessed emotional and behavioral changes and distress in the parents of 23 pediatric patients with epilepsy and the related neurocognitive and emotional comorbidities during the pandemic. In their study, they revealed that concerns for therapy monitoring and worries for a different contact with reference healthcare facilities were common. In a wider casuistry of children with special needs [22], parents of offspring with autism spectrum disorders were more likely to exhibit mental health issues compared to parents whose children had an intellectual disability or sensory impairments. Furthermore, behavioral problems of children and psychological demands of parents were common factors predicting poor parental mental health. Moreover, higher PCDI and parenting distress (PD) subscale scores of the PSI questionnaires were also associated with a diagnosis of autism spectrum disorder [22]. This latter evidence was also confirmed by another study [23] that showed a reciprocal influence between autism severity symptoms, parental distress and emotional well-being, along with a negative impact of missing healthcare support during pandemic.

To further explore the complex relationship between parental distress, offspring emotional and behavioral problems and quarantine-related factors during the COVID-19 outbreak, we conducted a cross-sectional study on a sample of parents of pediatric patients affected by neuropsychiatric conditions. In this study, we aimed to characterize the affected domains of parenting distress during lockdown and identify its clinical and demographic predictors.

## 2. Materials and Methods

The Strengthening the Reporting of Observational Studies in Epidemiology (STROBE) Statement guidelines [24] were in the reporting of this observational study. STROBE Checklist is available in Supplementary Table S1.

### 2.1. Recruitment and Data Collection

The present study was conducted by a research team of residents under the supervision of the Residency Director (RB) at the Department of Child and Adolescent Neurology and Psychiatry (University of Pisa, Italy) at the Stella Maris Foundation Hospital (IRCCS Stella Maris, Pisa, Italy) and was approved by the Tuscany Pediatric Ethics Committee (N. 95/20- 20.04.2020). This work is part of a larger project which aimed to investigate lockdown-related emotional and behavioral variations in terms of Child Behavior Checklist (CBCL) scores changes in pediatric neuropsychiatric population referring to IRCCS Stella Maris, during lockdown in April 2020 (for further details, please refer to Conti et al., 2020 [9]).

The online survey was comprehensive of PSI tool in order to correlate parental stress to information obtained from CBCL and EACD administration during lockdown (April–June 2020).

Residents contacted the parents of children who met the inclusion criteria via telephone and informed them about the aims and finalities of the study. Seventy-seven parents completed three questionnaires: the EACD socio-demographic questionnaire and the CBCL 1.5–5 years and CBCL 6–18 years were uploaded and shared on a dedicated online platform (“REDCap”, Research Electronic Data Capture, Vanderbilt University, Nashville, TN, USA); the PSI-4-SF questionnaire was instead filled in by parents directly on the Giunti Psychometrics testing platform to which they could access through a dedicated link. For further details on the online procedure of data collection, please refer to Conti et al., 2020.

Inclusion criteria were as follows: (i) age under 18 years; (ii) presence of neuropsychiatric disorders diagnosed at IRCCS Stella Maris according to multidisciplinary team (neurologist and psychiatrist, psychologist, speech therapist, educational therapist, and child therapist) and to a standardized assessment (Kiddie Schedule for Affective Disorders and Schizophrenia—Present and Lifetime [K-SADS-PL], Autism Diagnostic Observation Schedule—Second Edition [ADOS-2], Autism Diagnostic Interview—Revised [ADI-R], Child Behavior Checklist [CBCL], Griffith’s Developmental scale, Wechsler Intelligence Scales for Children [WISC], etc.) in the period between September 2019 and February 2020; further information on the recruitment process are available elsewhere (Conti et al., 2020); (iii) PSI completion on the Giunti Psychometrics testing platform during lockdown (April–June 2020); and (iv) the availability of CBCL and EACD scores obtained during lockdown (April–June 2020) on a devoted online platform (“REDCap”, Research Electronic Data Capture, Vanderbilt University, Nashville, TN, USA).

According to a comprehensive and multidimensional assessment approach used in clinical research in child and adolescent psychiatry and especially in our Hospital (see, for instance, the study by Cristofani C. et al. [25]), eight non-mutually exclusive diagnostic dimensions based on our clinical assessments, across several psychopathological, neurodevelopmental and neurological conditions, were defined in the present study. In particular, (1) internalizing (e.g., depression and anxiety) and (2) externalizing (e.g., oppositional defiant and conduct disorders) psychiatric disorders, (3) attention deficit hyperactivity disorder (ADHD), (4) language and speech sound disorders, (5) epilepsy and (6) cerebral palsy and other motor developmental disorders were coded as either present or absent. Conversely, (7) autism spectrum disorder (ASD) was classified according to symptoms severity into absent (I), subthreshold ASD condition or social communication disorder (II) and full-blown ASD (III), whereas (8) intellectual disability (ID) and/or developmental delay (DD) was classified into absent (I), borderline cognitive functioning (II) and overt ID/DD (III).

### 2.2. Measures

#### 2.2.1. Parental Stress Index

The Parental Stress Index questionnaire—fourth edition (PSI-4) is a test designed to identify parental stress, stressful parent–child relational systems, and therefore, the risk for the development of dysfunctional behaviors by both parents and children. It is intended for parents of children aged between one month and 10 years old. The test can be used as a screening and evaluation measure of the parenting system and to identify any kind of relational disorder that could lead to behavioral problems of both children and parents.

The short form of the PSI-4 questionnaire (PSI-4-SF), which was used in the current study, consists of 36 items which are divided into three subscales (Parental Distress (PD), Parent–Child Dysfunctional Interaction (PCDI) and Difficult Child (DC)) and a Total Stress (TS) scale. The PD scale (items from 1 to 12) refers to the level of distress that a parent is experiencing, due to their parental role; the PCDI scale (items from 13 to 24) is focused on the level of parental disappointment due to unmet expectations about the child and on the level of satisfaction perceived by the parent in the relationship with their child; the DC scale (items from 25 to 36) refers to difficulties in managing child’s dysfunctional behavior by the parent. Each item is rated on a five-point Likert scale (1 = strongly disagree, 2 = disagree, 3 = not sure, 4 = agree, 5 = strongly agree) with the three subscale scores ranging from 12 to 60. According to the normative tables for the Italian population, scores below the 85th percentile were considered within normal limits; scores within the 85th to 89th percentile indicate borderline parenting stress; scores within the 90th to 94th percentile indicate clinically significant parenting stress; and scores within the 95th to 100th percentile indicate clinically severe parenting stress. The TS score represents the overall level of parental stress, ranging from 36 to 180 and scores of 90 or above may be considered clinically relevant.

Internal consistency within each subscale of the PSI-4-SF questionnaire is high and validation studies conducted across several countries [26,27,28] suggest that this measure is a robust assessment tool that maintains its validity between non-English-speaking cultures.

#### 2.2.2. European Academy of Childhood Disability (EACD)

A socio-demographical questionnaire, purposely set up in collaboration with the European Academy of Childhood Disability (EACD), was anonymously completed by the enrolled families. This questionnaire was also distributed in the European study “EACD COVID-19 Survey-Families” for the evaluation of different aspects of daily life of young patients with neuropsychiatric disorders and their families during lockdown (http://edu.eacd.org/eacd-COVID-19-surveys-initial-report; March–May 2020). In the current study, only a limited number of items of the EACD General Questionnaire were used for statistical purposes, specifically related to the following subjects: the number of family unit members living together during lockdown (item 6a), the presence of outdoor spaces at home (item 6b), parent-rated quality (scored from 0 to 100) of the child relationship with teachers (item 11a) and peers (item 11b) during quarantine, parent-rated impact (scored from 0 to 100) of the COVID-19 outbreak on the child’s physical (item 12) and mental health (item 13) and financial repercussions (item 14), the availability of rehabilitation therapies during quarantine (item 16) and related subjective usefulness (scored from 0 to 100) perceived by parents (item 17a), parent-rated adjustment (scored from 0 to 100) of the offspring to lockdown measures (item 19) and related caregivers’ burden (scored from 0 to 100) (item 20), and changes in sleep habits (item 23) and autonomies of the child, as well as presence of current pharmacological treatments.

#### 2.2.3. Child Behavior Checklist

Child Behavior Checklist for children aged 1.5 to 5 years (CBCL 1.5–5) and Child Behavior Checklist for children aged 6–18 years (CBCL 6–18) are multiple item parent reports, the CBCL 1.5–5 is used for the evaluation of behavioral symptoms in preschoolers, whereas the CBCL 6–18 and to assess older child and adolescent psychopathology. Each item is rated on a three-point Likert scale (0, not true; 1, sometimes true; 2, very true). These questionnaires are composed of various scales which aimed to assess the child’s social, behavioral and emotional problems: three summary scales for both CBCL ½–5 and CBCL 6–18 (internalizing, externalizing and total problems), seven syndrome scales for CBCL ½–5 and eight syndrome scales for CBCL 6–18, five DSM-oriented scales for CBCL ½–5 and six DSM-oriented Scales for CBCL 6–18. The questionnaire consists of additional scales which, however, have not been considered in the present study.

Among the 77 patients enrolled, the CBCL–1.5/5 was used to enroll 34 preschoolers while the CBCL–6/18 was used to enroll 43 older children. For statistical analyses, only the three problems scales were initially retained and the T-scores from the two CBCL versions were merged together; further analyses were later performed using syndrome and DSM-oriented scales loading to the problems scale, which resulted in significant associations.

### 2.3. Statistical Analyses

The primary aim of the analyses was to identify, among the clinical and demographic variables listed above, potential factors that might influence parental stress, as assessed through the PSI-4-SF questionnaire subscale scores. Means and standard deviations (SD) were calculated for continuous variables. The Shapiro–Wilk test was used to assess the normality of the distributions of continuous dependent variables (i.e., PSI-4-SF questionnaire subscale scores) and either parametric or non-parametric analyses were performed accordingly. Univariate analyses were thus applied to identify statistical associations between dependent and independent variables as follows: either Student’s *t*-test or Mann–Whitney U test were carried out for binary independent variables; either analyses of variance (ANOVA) or Kruskal–Wallis tests were used for other categorical independent variables; either Pearson’s or Spearman’s rank correlation coefficients were estimated for continuous independent variables. Since univariate analyses implied the use of many tests of statistical significance, raising the problem of type I errors, this stage was only preliminarily applied to identify independent variables that could be retained as significant predictors and an uncorrected *p*-value level lower than 0.05 was still considered for this initial purpose.

Subsequently, selected independent variables were gathered in a backward stepwise generalized linear regression model for each PSI-4-SF questionnaire subscale. One variable was included or excluded from the model each time by comparing the Akaike Information Criterion (AIC) value, and the model with the lowest AIC was retained. The AIC value was, thus, used as a measure for evaluating the relative goodness-of-fit for linear regression models. Statistical significance was set at *p*-value < 0.05. Since the CBCL internalizing problems scale resulted in significant associations across all the regression models performed, syndrome and DSM-oriented scales loading to the CBCL internalizing problems scale were then retrieved and Spearman’s rank correlations were performed with the four PSI-4-SF subscales; *p*-value was corrected applying the Bonferroni method for multiple comparisons at the traditional significance level lower than 0.05.

Afterward, in order to examine the interplay among the variables that showed statistical significance with parental stress, one by-hand moderation and three multiple mediation models were performed using the Mediation package of RStudio^®^ software (PBC, Boston, MA, USA). Both direct and indirect effects of the EACD General Questionnaire-related variables on the PSI-4-SF subscales scores were assessed, with the possible mediation of the CBCL problems scales. The target models were tested, along with models inverting the mediator with the predictor and with a model inverting the mediator with outcome variables. The significance of the indirect effect was verified applying the bootstrapping method by Preacher and Hayes (2004) [29], which is particularly appropriate for small samples, and unstandardized indirect effects were computed for each of 1000 bootstrapped samples; 95% confidence interval was estimated by determining the indirect effects at the 2.5th and 97.5th percentiles. Statistical analyses were performed using RStudio^®^ software.

## 3. Results

### 3.1. The Sample

Among the 77 children, 66 were males (85.71%) and mean age was 6.62 ± 3.12 years old. The patients enrolled presented complex clinical diagnoses with multiple comorbidities. In our children group, 19 (24.68%) presented externalizing psychiatric disorders and 25 (32.47%) internalizing psychiatric disorders, 28 (36.36%) were affected by ADHD, 40 (51.95%) presented a language and/or speech sound disorders, 4 (5.19%) children were affected by epilepsy and 8 (10.39%) by cerebral palsy or other motor developmental conditions. Seventeen (22.08%) patients were affected by overt ID/DD, while 19 (24.68%) presented a borderline intellectual functioning. Forty-seven (61.04%) children were diagnosed with complete ASD, whereas 13 (16.88%) had a sub-threshold ASD condition or a social communication disorder. All of the 77 patients’ parents enrolled fulfilled the questionnaires, of these, 25 were fathers (32.47%) and mean age was 42.53 ± 6.87 years old.

### 3.2. PSI-4-SF Questionnaire

#### 3.2.1. Total Stress Subscale (TS)

Univariate analyses identified statistically significant associations between TS subscale scores and the following independent variables: parents’ gender, pharmacological treatment, availability of rehabilitation therapies and perceived usefulness, changes in sleep habits and autonomies, relationship quality with teachers and peers, offspring adjustment to lockdown, and the CBCL internalizing and externalizing problems subscales. The described significantly independent variables were then gathered in a backward stepwise generalized linear regression model with the TS subscale of PSI-4-SF questionnaire as dependent variable.

The final model with the lowest AIC goodness-of-fit value of 564.96 included the following independent variables: parents’ gender, relationship quality with peers, availability of rehabilitation therapies and the CBCL internalizing and externalizing problems subscales (see Table 1A). A significant positive association was finally found between the TS subscale of PSI-4-SF questionnaire and the CBCL internalizing problems scale, while a nearly significant positive association was identified with the CBCL externalizing problems scale.

As shown in Table 2, Spearman’s rank correlations identified significant positive associations between the PSI-4-SF TS subscale and all the CBCL subscales loading to the internalizing problems scale, namely the DSM-oriented Affective Problems and Anxiety problems subscales, the anxious/depressed, the withdrawn/depressed and the somatic -problems symptoms subscales.

#### 3.2.2. Parental Distress Subscale (PD)

Statistically significant associations were identified between PD subscale scores and the following independent variables: parents’ age and gender, financial repercussions, the availability of rehabilitation therapies, number of siblings, changes in sleep habits, relationship quality with teachers, caregivers’ burden and the CBCL internalizing and externalizing problems subscales, with the univariate analyses. The described significantly independent variables were then gathered in a backward stepwise generalized linear regression model with the PD subscale of PSI-4-SF questionnaire as a dependent variable.

Therefore, the final model with the lowest AIC goodness-of-fit value of 469.73 included the following independent variables: parents’ gender, number of siblings, caregivers’ burden and the CBCL internalizing and externalizing problems subscales (see Table 1B). Moreover, a significant positive relationship was found between the PD subscale of PSI-4-SF questionnaire and the CBCL internalizing problems scale, and a significant association emerged between the PSI-4-SF subscale and parents’ gender, with fathers displaying lower PD scores than mothers.

As shown in Table 2, Spearman’s rank correlations identified significant positive associations between the PSI-4-SF PD subscale and some of the CBCL subscales of the internalizing problems scale, namely the DSM-oriented affective problems, anxiety problems subscales, and the anxious/depressed symptoms subscales.

We then tested whether the parents’ gender affected the direction or strength of the significant association between the CBCL internalizing problems and the parent distress. The regression coefficient of 0.2044 for the interaction between parents’ gender and CBCL internalizing problems is not statistically significant, thus no significant moderation effect can be hypothesized.

#### 3.2.3. Parent–Child Dysfunctional Interaction Subscale (PCDI)

Univariate analyses identified statistically significant associations between PCDI subscale scores and the following independent variables: diagnoses of ASD and epilepsy, the availability of rehabilitation therapies and related perceived usefulness, pharmacological treatments, changes in autonomies, children’s age, relationship quality with teachers and peers, offspring adjustment to lockdown measures and the CBCL internalizing and externalizing problems scales. The described significantly independent variables were then gathered in a backward stepwise generalized linear regression model with the PCDI subscale of PSI-4-SF questionnaire as the dependent variable.

The final model with the lowest AIC goodness-of-fit value of 413.16 included the following independent variables: diagnoses of ASD and epilepsy, relationship quality with teachers, perceived usefulness of rehabilitation therapies, and the CBCL internalizing and externalizing problems scales (see Table 1C). A significant positive association was found between the PCDI subscale of PSI-4-SF questionnaire and the CBCL internalizing problems scale, while a significant negative association was found with the relationship quality with teachers.

As shown in Table 2, Spearman’s rank correlations identified significant positive associations between the PSI-4-SF PCDI subscale and some of the CBCL subscales of the internalizing problems scale, namely the DSM-oriented affective problems, anxiety problems subscales, and the anxious/depressed symptoms subscales.

**Figure 1:**
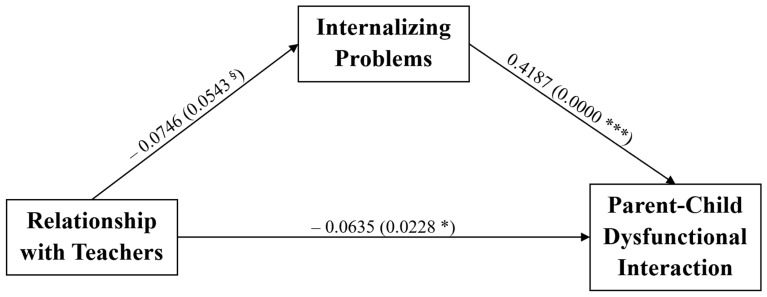
Multiple mediation model on the association between the relationship quality with peers, internalizing problems and the PSI-4-SF parent–child dysfunctional interaction subscale. The effect of the children’s relationship with teachers on the parent–child dysfunctional interaction is mediated via the internalizing problems of the child. ^§^ 0.05 < *p*-value < 0.06; * *p*-value < 0.05; *** *p*-value < 0.001.

Subsequently, we tested a multiple mediation model to examine the possible mediation role of the CBCL internalizing problems on the negative association found between the relationship quality with teachers and the PSI-4-SF PCDI subscale. Specifically, the effect of the children’s relationship with teachers on the parent–child dysfunctional interaction was mediated via the internalizing problems of the child (see Figure 1), against the models inverting the mediator with the predictor and the mediator with outcome variables (data not shown). Both regression coefficients between relationship with teachers and PCDI and between CBCL internalizing problems and PCDI were significant. Furthermore, the indirect effect assessed through an unstandardized bootstrapping procedure was statistically significant, as shown in Table 3A.

#### 3.2.4. Difficult Child Subscale (DC)

Univariate analyses identified statistically significant associations between DC subscale scores and the following independent variables: diagnoses of ADHD and other externalizing psychiatric disorders, parents’ gender, presence of grandparents and number of other family members, impact on child physical and mental health, pharmacological treatments, changes in autonomies, relationship quality with peers, children’s adjustment to lockdown, and the CBCL externalizing and internalizing problems scales. The described significantly independent variables were then gathered in a backward stepwise generalized linear regression model with the DC subscale of PSI-4-SF questionnaire as a dependent variable.

The final model with the lowest AIC goodness-of-fit value of 405.23 included the following independent variables: a diagnosis of externalizing psychiatric disorder, parents’ gender, relationship quality with peers, and the CBCL externalizing and internalizing problems scales (see Table 1D). A significant positive association was found with both the internalizing and the externalizing problems scales of the CBCL questionnaire; whereas a significant negative association was found between the DC subscale of the PSI-4-SF questionnaire and the relationship quality with peers.

As shown in Table 2, Spearman’s rank correlations identified significant positive associations between the PSI-4-SF DC subscale and all the CBCL subscales belonging to the internalizing problems scale, namely the DSM-oriented affective problems and anxiety problems subscales, the anxious/depressed, the withdrawn/depressed and the somatic problems symptoms subscales.

**Figure 2:**
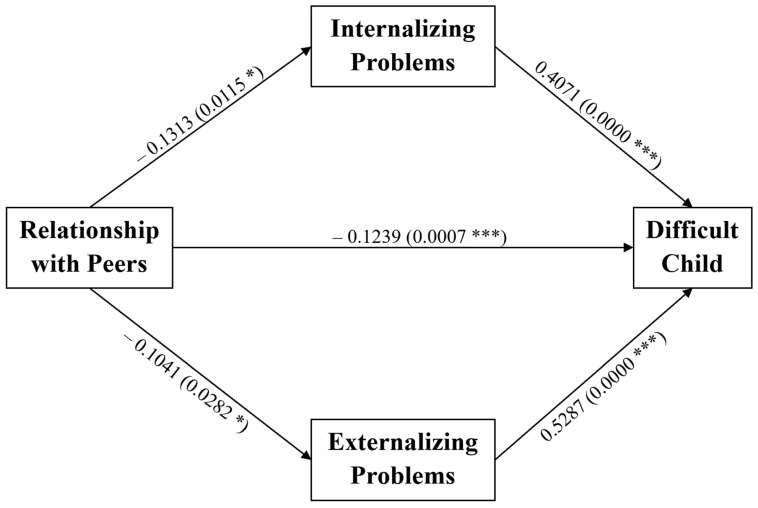
Multiple mediation model on the association between the relationship quality with peers, internalizing and externalizing problems and the PSI-4-SF Difficult Child subscale. The effect of the children’s relationship with peers on the Difficult Child subscale is mediated both via the internalizing and externalizing problems of the child. * *p*-value < 0.05; *** *p*-value < 0.001.

Thereafter, we tested a multiple mediation model to examine the possible mediation role of the CBCL internalizing and externalizing problems on the negative association between the relationship quality with peers and the PSI-4-SF DC subscale. Specifically, the effect of the children’s relationship with peers on the Difficult Child subscale was mediated both via the internalizing and externalizing problems of the child (see Figure 2), against the models inverting the mediator with the predictor and the mediator with outcome variables (data not shown). Both regression coefficients between relationship with peers and DC and between CBCL internalizing and externalizing problems and DC were significant. Furthermore, the indirect effect assessed through an unstandardized bootstrapping procedure was statistically significant, as shown in Table 3B,C.

**Table 1:** PSI-4-SF questionnaire: backward stepwise generalized linear models.

**A. TS Subscale**	**Estimate**	**SE**	**t-Value**	***p*-Value**
Intercept	20.5482	12.8649	1.597	0.1152
Parents’ Gender	5.7494	3.6470	1.576	0.1199
Relation with Peers	−0.1164	0.0728	−1.599	0.1149
Available Therapy	−6.7751	3.8613	−1.755	0.0842
CBCL Internalizing	0.8623	0.2011	4.287	0.0001 ***
CBCL Externalizing	0.4452	0.2232	1.995	0.0504 ^§^
AIC Goodness-of-Fit Value	564.96			
**B. PD Subscale**	**Estimate**	**SE**	**t-Value**	***p*-Value**
Intercept	6.2035	6.4662	0.9590	0.3411
Parents’ Gender	4.4275	1.8406	2.4052	0.0192 *
Siblings (n = 1)	2.5406	1.8332	1.3866	0.1707
Siblings (n ≥ 2)	−4.1015	2.6942	−1.5224	0.1330
Available Therapy	−2.6682	1.9212	−1.3894	0.1699
Parents’ Burden	0.0757	0.0464	1.6312	0.1081
CBCL Internalizing	0.2785	0.0832	3.3490	0.0014 **
AIC Goodness-of-Fit Value	469.73			
**C. PCDI Subscale**	**Estimate**	**SE**	**t-Value**	***p*-Value**
Intercept	6.8569	5.6457	1.2150	0.2295
ASD (Subthreshold)	3.6202	2.1212	1.7072	0.0932
ASD (Full-blown)	−2.4952	1.6842	−1.4823	0.1439
Epilepsy Diagnosis	3.8175	2.8132	1.3576	0.1800
Relation with Teachers	−0.0443	0.0213	−2.0798	0.0421 *
Therapy Usefulness	−0.0372	0.0227	−1.6401	0.1065
CBCL Internalizing	0.2913	0.0793	3.6747	0.0005 ***
CBCL Externalizing	0.1720	0.0941	1.8283	0.0728
AIC Goodness-of-Fit Value	413.16			
**D. DC Subscale**	**Estimate**	**SE**	**t-Value**	***p*-Value**
Intercept	3.2373	4.9220	0.6581	0.5132
Externalizing Psych	2.9013	1.4665	1.9780	0.0525 ^§^
Parents’ Gender	2.2257	1.3265	1.6782	0.0986
Relation with Peers	−0.0797	0.0270	−2.9465	0.0046 **
CBCL Internalizing	0.1726	0.0727	2.3745	0.0208 *
CBCL Externalizing	0.3546	0.0923	3.8409	0.0003 ***
AIC Goodness-of-Fit Value	405.23			

Note: PSI-4-SF: Parental-Stress-Index-4-short form; TS Subscale: Total Stress Subscale; PD subscale: Parental Distress subscale; PCDI Subscale: Parent–Child Dysfunctional Interaction Subscale; DC Subscale: Difficult Child Subscale; *n*: Number; CBCL: Child Behavior Checklist; ASD: Autism Spectrum Disorder; SE; Standard Error. ^§^ 0.05 < *p*-value < 0.06; * *p*-value < 0.05; ** *p*-value < 0.005; *** *p*-value < 0.001.

**Table 2:** Spearman’s rank correlations: CBCL internalizing problems subscales and PSI-4-SF subscales.

**A. *p*-Values**	**TS**	**PD**	**PCDI**	**DC**
Affective Problems	0.0000 ***	0.0000 ***	0.0000 ***	0.0000 ***
Anxiety Problems	0.0000 ***	0.0016 **	0.0000 ***	0.0000 ***
Anxious/Depressed	0.0000 ***	0.0005 ***	0.0000 ***	0.0000 ***
Withdrawn/Depressed	0.0005 **	0.0069	0.0052	0.0016 **
Somatic Problems	0.0004 **	0.0064	0.0103	0.0005 ***
**B. Rho Coefficients**	**TS**	**PD**	**PCDI**	**DC**
Affective Problems	0.6877	0.4721	0.6726	0.6059
Anxiety Problems	0.5723	0.3766	0.4908	0.5843
Anxious/Depressed	0.5685	0.4111	0.5000	0.5362
Withdrawn/Depressed	0.4117	0.3266	0.3375	0.3772
Somatic Problems	0.4169	0.3293	0.3113	0.4124

Note: PSI-4-SF: Parental-Stress-Index-4-short form; TS Subscale: Total Stress Subscale; PD subscale: Parental Distress subscale; PCDI Subscale: Parent–Child Dysfunctional Interaction Subscale; DC Subscale: Difficult Child Subscale. ** *p*-value < 0.005; *** *p*-value < 0.001.

**Table 3:** PSI-4-SF questionnaire: by-hand moderation model and multiple mediation models.

**A. PCDI Subscale**	**Estimate**	**95% CI–Lower**	**95% CI–Upper**	***p*-Value**
ACME	−0.0312	−0.0727	0.0000	0.0281 *
ADE	−0.0305	−0.0800	0.0200	0.2260
Total Effect	−0.0618	−0.1182	−0.0100	0.0342 *
Prop Mediated	0.5056	0.0349	1.6600	0.0423 *
**B. DC (Internalizing)**	**Estimate**	**95% CI–Lower**	**95% C–Upper**	***p*-Value**
ACME	−0.0535	−0.1113	−0.0200	0.0000 ***
ADE	−0.0841	−0.1485	0.0000	0.0561 ^§^
Total Effect	−0.1376	−0.2005	−0.0601	0.0000 ***
Prop Mediated	0.3886	0.1219	1.0552	0.0000 ***
**C. DC (Externalizing)**	**Estimate**	**95% CI–Lower**	**95% CI–Upper**	***p*-Value**
ACME	−0.0550	−0.1025	−0.0100	0.0220 *
ADE	−0.0825	−0.1280	−0.0431	0.0000 ***
Total Effect	−0.1376	−0.2011	−0.0725	0.0041 **
Prop Mediated	0.4001	0.1157	0.6301	0.018 *

Note: PSI-4-SF: Parental-Stress-Index-4-short form; TS Subscale: Total Stress Subscale; PD subscale: Parental Distress Subscale; PCDI Subscale: Parent–Child Dysfunctional Interaction Subscale; DC Subscale: Difficult Child Subscale; ACME: Average Causal Mediation Effects; ADE: Average Direct Effect; Prop Mediated: Proportion Mediated; CI: Confidential Interval. ^§^ 0.05 < *p*-value < 0.06; * *p*-value < 0.05; ** *p*-value < 0.005; *** *p*-value < 0.001.

## 4. Discussion

During the COVID-19 outbreak and the related national quarantine, prolonged school closure, home confinement and increase in smart-working often required that parents play the role of caregivers and hard-workers at the same time. If a negative psychological impact of such lockdown measures was reported on the general adult population [30,31], the most severe psychological impact of lockdown measures was found in children with special needs or disabilities and their families [32]. Our study suggests that it is of great importance to include measures of parenting stress when assessing children with disabilities in stressful situations. In this perspective, several previous studies confirmed that assessing parental stress may also have treatment implications since it is an important target variable for both prevention and intervention [33,34].

Our study confirms the psychological impact on families with children with neuropsychiatric disabilities, bringing out, as the most striking result, a significant positive correlation between internalizing symptoms of our pediatric sample and parental distress. Specifically, these results emerged by exploring the associations among their daily life routines during the lockdown, CBCL internalizing and externalizing problems, clinical diagnoses, and the different scales of PSI-SF questionnaire. The correlation between internalizing problems and parental stress may be related to a particular difficulty of parents to understand their children’s symptoms and their consequent sense of powerlessness and helplessness. Therefore, it might be useful to explain to parents the importance of paying attention to the internalizing symptoms as an expression of a child discomfort. Interestingly, Costa and colleagues (2006) [35] found a specific association between child and adolescent internalizing symptoms and the PCDI scale of the PSI-SF questionnaire. Indeed, the authors argued that such specificity might be related to parents’ unmet expectations of their children, or to a non-reinforcing reciprocal interaction. It was therefore assumed that the negative parental beliefs, listed above, may prompt withdrawn, anxious, or depressed behaviors in children [35]. In addition, anxious or depressed children may also significantly escalate parental stress levels, increasing the likelihood of inadequate parenting behaviors such as overbearing.

During quarantine, parents were alone in supporting their children doing homework and promoting a positive development and new learning experiences to their children [7]. Spinelli and colleagues, in their recent study, highlighted that the parental perception of the difficulties related to the COVID-19 lockdown increased parenting stress levels, raising emotional and behavioral difficulties in children [17].

We hypothesize that this situation might have increased parental anxiety over parenting expectations, and anxious parents may be over-stressed and worried. This could have led to overcontrolling or inconsistent parenting style, which might have increased children’s anxiety [23].

Several other studies also point out a strong link between parenting stress and child psychopathology. For example, in a pioneering cross-cultural study, child internalizing symptoms across cultures were significantly associated with maternal parenting stress, as independently reported by mothers and children [36].

In our opinion, clinicians should primarily support caregivers to recognize and accept internalizing symptoms of children; furthermore, it could be useful to pay more attention to families vulnerable to the social aspects of the crisis, such as the loss of teaching support. It is of interest that, while in American studies (COVID-19 and Parent–Child Psychological Well-Being), parental stress was found to correlate with socio-economic class, job insecurity and economic loss, this aspect did not emerge in our study. These data could be linked to the different organization of the Italian health system, in which health care is guaranteed to all families, regardless of socio-economic status, underlining the importance of a public health system on the psychophysical well-being of the population.

Moreover, we found a significant negative association between the quality of the relationship of children with their teachers and the PCDI scale of the PSI-SF questionnaire, which focuses on the level of parental disappointment due to unmet expectations of their child and on the level of satisfaction perceived by the parent in the relationship with their child. Furthermore, the effect of the children’s relationship with teachers on the parent–child dysfunctional interaction was likely influenced by the internalizing problems of the child. In this regard, it is important to underline the impact of environmental factors on parental stress, both positively and negatively. In fact, distance learning could have caused a deterioration in the teachers–child relationship. Online environments could have caused feelings of anonymity, reducing participation and increasing the withdrawal of children. We might speculate that, without face-to-face contact, teachers might have not noticed the students’ non-verbal and behavioral cues which may indicate that children are struggling or disengaged. In addition, the child might have found it more difficult to express any concerns, doubts or worries, with increased anxiety symptoms and a lack of self-esteem, of academic self-esteem, and express depressive symptoms. We could also suppose that a child with internalizing problems could show more inhibition in answering an online lesson through webcams, reflecting less participation to the lesson and a loss of quality in their relationship with teachers.

A significant negative association was found between the DC subscale of the PSI-4-SF questionnaire and the relationship quality with peers. Moreover, in the DC scale, which refers to difficulties in managing child’s dysfunctional behavior by the parent, a significant negative association was found with the relationship quality with peers and a significant positive association was found with both the internalizing and the externalizing problems scales of the CBCL questionnaire. Interestingly, the association between DC scale and CBCL internalizing and externalizing symptoms, was already described by Costa and colleagues (2006) in their study conducted on a clinical sample. Regarding peer-to-peer interaction, during a time when all face-to-face interactions were limited, and most of them had to be online, we can suppose that having a psychiatric issue increased the difficulty of parents to manage their children without the support of small neighborhood communities, increasing the burden of caregivers and parental stress. In this regard, it is mandatory to emphasize the role of socialization with peers as a protective factor against parental stress. Therefore, it may be useful to take into account, in future therapeutic decisions, the possible benefit of group rather than individual rehabilitation interventions.

Interestingly, we found a parental gender difference in the DC score of the PSI. In particular, fathers scored lower than mothers on this scale. This finding could be in line with what was recently reported in the study by Bikmazer et al. (2020) [14] which found that the gender (woman) of the parent and the younger age of the parents are independently associated with increased parental stress. In line with our results, Petrocchi et al. (2020) found that mothers with reduced coping skills and higher stress levels were more likely to attribute negative emotions to their children compared to their positive emotions.

Moreover, no correlation between parental stress and ASD and epilepsy was found in the PCDI and PD subscale scores of the PSI questionnaire. However, a preliminary statistically significant association was found in the univariate analysis for the PCDI subscale scores. This finding partially matches with what is reported by Chen et al. (2020) [22] and Alhuzimi et al. (2021) [23], who both used the PSI questionnaire to assess parental distress, showing a reciprocal influence between autism severity symptoms, parental distress and emotional well-being, along with a negative impact of missing healthcare support during pandemic. However, these previous studies were conducted in China and in Saudi Arabia in a larger sample of patients, so we can suppose that only a preliminary statistically significant association was found due to the differences of Italian social fabric and the heterogeneous sample size taken into account.

The results of the present study can be reliably generalized to the Italian neuropsychiatric population since statistical errors were avoided through accurate statistical analyses including Bonferroni’s correction method. Nonetheless, some limitations of the current study are worthy of discussion, namely a possible selection bias. Indeed, a small sample size was taken into account. Moreover, the population enrolled was not homogenous, being mostly composed of male and patients with psychiatric symptoms; only 12 children were affected by neurological conditions (epilepsy and CP). However, to provide an overview of the parental distress of the neuropsychiatric population referred to our institute, no prior hypothesis on the sample was made, resulting in a heterogeneous sample of parents who fulfilled the questionnaire. Moreover, the incidence of psychiatric disorders in children is more than the incidence of neurological conditions, such as cerebral palsy or epilepsy, so a wider sample, more representative of the neuropsychiatric population, was considered [37,38,39]. Furthermore, the respondent sample was also heterogeneous, with only 32% of respondents being fathers. Another limitation should be addressed; in our study, we assumed that there was a positive correlation between parental stress and internalizing problems due to the COVID-19 lockdown. This hypothesis was supported by recent literature data [17]; however, the actual impact could have been detected if an evaluation of parental stress had been made before quarantine.

## 5. Conclusions

Our study highlighted several clinical implications that could be addressed in Italy and other countries when dealing with the COVID-19 pandemic and the related restrictions. In particular, our study identified a significant positive correlation between the internalizing symptoms of our pediatric sample and parental distress, pointing out the importance of supporting parents in stressful situations when their child presents internalizing symptoms.

A negative association was found between the quality of the relationship of children with their teachers and the scores at the PCDI scale. On the basis of this, it could be useful for clinicians to keep in mind to investigate the quality of the relationship between children and their teachers and its possible correlation with self-esteem. Moreover, due to the significant negative association found between the scores at the DC subscale and the relationship quality with peers, it is important to pay attention to the role of socialization with peers as a protective factor against parental stress.

## Data Availability

The data presented in this study are available on request from the corresponding author. The data are not publicly available due to restriction for privacy.

## Supplementary Materials

The following are available online at https://www.mdpi.com/article/10.3390/ijerph18157902/s1, Table S1: STROBE Statement—checklist of items that should be included in reports of observational studies.
